# Quantitative Analysis by 3D Graphics of Thoraco-Abdominal Surface Shape and Breathing Motion

**DOI:** 10.3389/fbioe.2022.910499

**Published:** 2022-07-13

**Authors:** Andrea Aliverti, Davide Lacca, Antonella LoMauro

**Affiliations:** Dipartimento di Elettronica, Informazione e Bioingegneria, Politecnico di Milano, Milano, Italy

**Keywords:** chest wall, opto-electronic plethysmography, breathing, volume, shape, kinematics

## Abstract

Chest wall motion can provide information on respiratory muscles' action and on critical vital signs, like respiration and cardiac activity. The chest wall is a structure with three compartments that are independent to each other and can move paradoxically according to the pathophysiology of the disease. Opto-electronic plethysmography (OEP) allows for non-invasively 3D tracking of body movements. We aimed to extend the characteristics of OEP analysis to local analyses of thoraco-abdominal surface geometry and kinematics during respiration. Starting from the OEP output file, the 3D markers’ coordinates were combined with a triangulation matrix. A smoothing procedure (an automatic and iterative interpolation process to increase the number of vertices from 93 to 548) was applied to allow for precise local analysis of the thoraco-abdominal surface. A series of measurements can be performed to characterize the geometry of the trunk and its three compartments, in terms of volumes, height, diameters, perimeters, and area. Some shape factors, such as surface-to-volume ratio or height-to-perimeter ratio, can be also computed. It was also possible to build the vector field associated with the breathing motion of all the vertices, in terms of magnitude and motion direction. The vector field data were analyzed and displayed through two graphic tools: a 3D heatmap, in which the magnitude of motion was associated to different colors, and a 3D arrow plot, that allowed us to visualize both the magnitude and the direction of motion with color-coded arrows. The methods were applied to 10 healthy subjects (5 females) and also applied to two cases: a pregnant woman at each trimester of gestation and a patient before and after a demolition thoracic surgery. The results proved to be coherent with the physiology of healthy subjects and the physiopathology of the cases. We developed a new non-invasive method for respiratory analysis that allowed for the creation of realistic 3D models of the local and global trunk surface during respiration. The proposed representation constituted a very intuitive method to visualize and compare thoraco-abdominal surface movements within and between subjects, therefore enforcing the potential clinical translational value of the method.

## Introduction

The chest wall is defined as all tissues, comprising skin, fat, muscles, and the thoracic skeleton, that form a protective structure around vital organs in the area between the neck and the abdomen. The interconnection between the thoracic skeleton and the muscular components forms a dynamic structure able to expand and shrink, thereby changing the intrathoracic volume and allowing for breathing to take place.

The interest in measuring the shape, the motion, the distortion as well as in modeling the chest wall started in the late 60s (Konno and Mead, 1967). All this interest arose because chest wall motion can provide information on respiratory muscles' action and on critical vital signs, like respiration and cardiac activity. The way chest wall motion was assessed changed over time. The first studies were based on measurements of distinct punctual displacements with magnetometers (Mead et al., 1967) and then areas were measured through inductive plethysmography (Lumb and Nunn, 1991). However, these methods supplied limited information. Magnetometers did not provide simultaneous measurements for more than three or four diameters while inductive plethysmography provides changes in only a single rib cage or abdominal cross-section.

The three-dimensional assessment of motion and shape within each thoraco-abdominal compartments was therefore lacking until opto-electronic plethysmography (OEP) was introduced (Cala et al., 1996). Thanks to this technique, the chest wall passed from being studied as a structure with two compartments (or degrees of freedom), the rib cage and the abdomen (Konno and Mead, 1967), to a structure with three compartments, namely the pulmonary ribcage, the abdominal ribcage and the abdomen Macklem et al. (1983) and Ward et al. (1992).

OEP is a non-invasive measurement technique, based on an automatic motion analyzer system that provides 3D tracking of body movements. The OEP system uses a set of infrared charge-coupled device cameras to capture the 3D motion of reflective passive markers placed on the skin of subjects according to precise anatomical reference points. The system also permits the chest wall volume variations that can be calculated from markers’ positions through surface triangulation and successive integration. OEP can capture the overall motion of the chest wall with great precision since it uses a high number of markers (typically 89 for sitting or standing position) that entirely cover the thoraco-abdominal surface.

**FIGURE 1:**
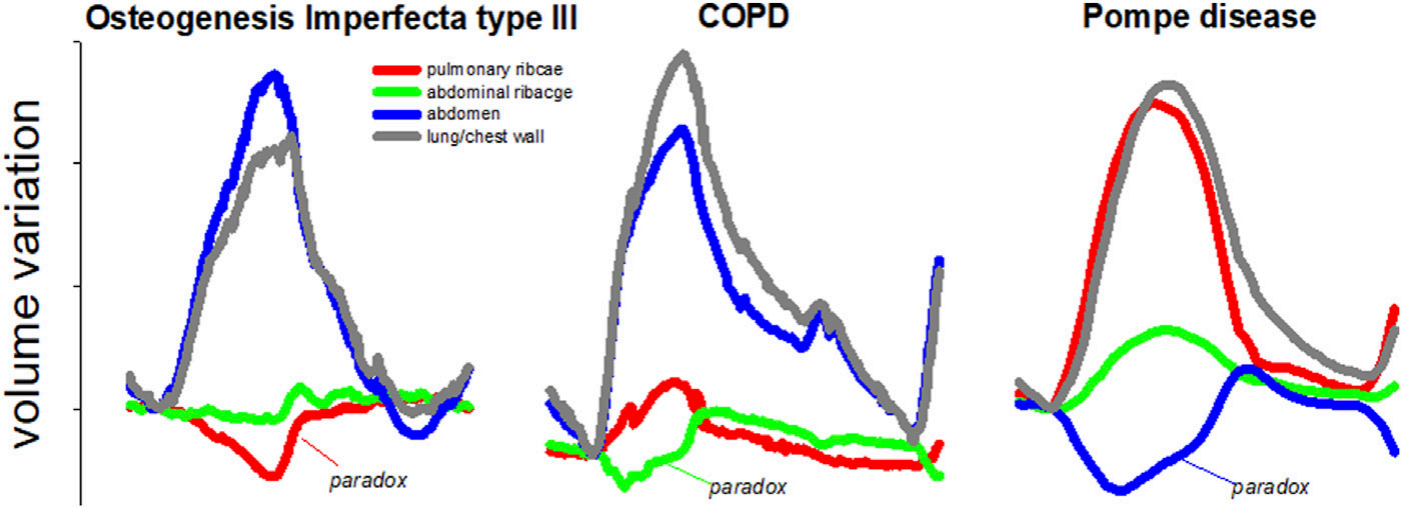
Representative breaths during resting breathing in three different pathologies: Osteogenesis Imperfecta type III (left panel); chronic obstructive pulmonary disease (middle panel) and glycogen storage disease type 2 (right panel). The volume of the total chest wall (grey line) as well as of its three compartments, namely pulmonary ribcage (red line); abdominal ribcage (green line), and abdomen (blue line) are presented. The paradox of inspiratory inward movement, defined as volume reduction, is also indicated for each pathology. These are original images of data previously published in papers dedicated to Osteogenesis Imperfecta (LoMauro et al., 2012); chronic obstructive pulmonary disease (Wilkens et al., 2010), and glycogen storage disease type 2 (Remiche et al., 2013).

Despite the high accuracy, respiratory analysis with OEP can only focus on the global behavior of the chest wall and/or of its three compartments at most (Cala et al., 1996; Romei et al., 2010). It is of clinical importance to study breathing not only in terms of global volume variation, but also to focus on compartmental volume variations. Each compartment is independent to each other and can move paradoxically according to the pathophysiology of the disease. For example, as shown in Figure 1, Osteogenesis Imperfecta is characterized by paradoxical inward inspiratory motion of the pulmonary ribcage (LoMauro et al., 2012). Osteogenesis Imperfecta is a rare genetic disorder of collagen production leading to skeletal deformities (Rauch and Glorieux, 2004), including the ribcage with an important impact on breathing function, characterized by hypoventilation and paradoxical breathing, particularly in the most severe non-lethal type III form (LoMauro et al., 2012). Diaphragmatic paralysis is evident when the paradoxical inward inspiratory motion of the abdomen occurs (LoMauro et al., 2021). This was the case of glycogen storage disease type 2, an autosomal recessive metabolic disorder that damages muscle and nerve cells throughout the body, including the diaphragm (Remiche et al., 2013).

Finally, in pulmonology, the Hoover sign is defined as the paradoxical inward inspiratory motion of the abdominal ribcage in severe chronic obstructive pulmonary disease patients. This is an important clinical sign of poor prognostic information in patients with airway obstruction and it can serve to complement other clinical or functional tests (Johnston et al., 2008; Wilson, 2015).

What is missing is the ability to provide information about local kinematics and therefore shift the point of view from the whole trunk to single points on the surface.

For these reasons, we aimed to improve and extend the characteristics of OEP analysis, by developing and providing useful tools to perform local analyses of thoraco-abdominal surface geometry and kinematics during respiration. More in detail, we aimed to develop and implement a method that, starting from the standard OEP measurement protocol based on 89 markers positioned in defined anatomical landmarks, could allow an enhanced breathing kinematic analysis able to: 1) quantify the local displacement of the chest wall during breathing by interpolating the original dataset (89 markers) and meshing the thoracoabdominal surface; 2) be reproducible, that is, to allow the comparison of data within subjects (e.g., under different conditions, maneuvers and/or lung volumes) and between subjects (e.g., males vs. females, healthy vs. pathological subjects, etc.) and to create a map of statistical significance of differences in kinematics.

## Materials and Methods

### Models Creation

The creation of the 3D models of the trunk started from the output file of the OEP acquisition. This file is called the TDF file and contains the three-dimensional coordinates of the markers over time. First, the 3D markers’ coordinates were extracted to generate the coordinates matrix. Four extra virtual markers needed to be firstly computed and added to properly close the trunk shape: one at the top, one at the bottom, and two on the sides. These markers had to be added ex-post because the presence of the head and the limbs, which did not allow to define the trunk’s boundaries during the acquisition. The extra markers’ locations were computed by averaging the position of a set of already existing markers. In detail, the extra markers on the top and the bottom were the mean of all the markers in the highest and the lowest lines of both frontal and posterior sides, respectively. Similarly, the extra markers on two sides were the mean of all the markers in the more lateral right and left lines of both frontal and posterior sides. Since we were interested not only in the total trunk shape but also in its three compartments, different extra markers needed to be defined to close each of the corresponding shapes. Similarly, for each compartment, the extra markers were the mean of the markers of the highest, of the lowest, of the most right lateral, and of the most left lateral lines in both frontal and posterior sides. Given the 3D coordinates of the whole trunk, three reference levels along the longitudinal direction, corresponding to three anatomical reference points (namely, angle of Louis, xiphoid, and umbilicus) were then computed. These points were mapped to specific markers and their position was obtained by averaging the coordinates of the markers on the same level. The reference levels were generated and saved by running specific scripts in MATLAB (MathWorks® Inc.). Once the coordinates matrix was complete, the 3D shapes were created through another script in which the links between markers were specified as a triangulation matrix. By combining these two matrices it was possible to define and export 3D meshes as STL files. The 3D models were then imported in 3ds Max 2020 (Autodesk®, San Rafael, CA, United States) to be optimized. This software is a professional piece of software for 3D modeling and computer graphics, which allowed for direct manipulation, visualization, and measurement of the trunk models. 3ds Max offers a free academic version of the software that includes all the features to manage the 3D models. This software was selected because it features a series of built-in tools that analyze the trunk models faster and less complex. However, starting from the markers’ coordinates, all the processes that will be described next and that take place in 3ds Max can be repeated by applying standard computational algorithms in any other environment. The first step in 3ds Max was to check if the imported models presented any errors, such as welded vertices, flipped normals, or holes in the mesh. Also, the models’ scale was increased by a factor of 100 to obtain more accurate measurements and to match realistic dimensions. After this pre-processing, a smoothing procedure was applied to the models. The smoothing consisted in applying a “modifier” (that worked as a filter) to the models, which increased the number of vertices of the mesh, making it more precise and realistic. The modifier used is called TurboSmooth. It applies a fast, memory-efficient, and iterative smoothing procedure to the selected geometry. More in detail, it uses a single smoothing method (NURMS) that can be applied only to an entire object without sub-object levels with a triangle-mesh object as outputs. TurboSmooth allows the subdivision of the geometry while interpolating the angles of new faces at corners and edges. It also applies a single smoothing group to all faces in the object. The effect of TurboSmooth is to round over corners and edges as if they had been filed or planed smooth to produce a Non-Uniform Rational MeshSmooth object (NURMS), being similar to a NURBS object with the possibility to set different weights for each control vertex. The overall effect is therefore a more curved and complex mesh. After selecting one or more 3D models in the scene, the smoothing process is applied automatically by 3ds Max when the user selects the TurboSmooth modifier in the tools menu. Since all the models started with the same number of vertices, the smoothing was applied in the same way for all the models, making the resulting versions consistent. This means that TurboSmooth computed and placed the same number of new vertices in the same way for all the models to which it was applied. The original vertices (i.e., the markers) were preserved after the smoothing so that they could always be used as references. The results in terms of vertices increase and average percentage volume reduction are reported in the following table:

**Table:** 

# Of iterations	# Of vertices	Avg. volume reduction %
0	93	0
**1**	**548**	**4.470**
2	2,186	1.329
3	8,738	0.337
4	34,946	0.085
5	139,778	0.025
6	559,106	0.048

Only one iteration was applied (bold values in table). In this way, the number of vertices was increased enough to obtain a more realistic mesh but at the same time, it was low enough to manage computations for every vertex (for example the calculation of the vector field, that is based on distances between corresponding vertices). Also, the first iteration causes a higher volume reduction and after that it is not as significant. Moreover, 3ds Max computational times slow down as the iterations increase because of the rendering of such complex meshes.

**FIGURE 2:**
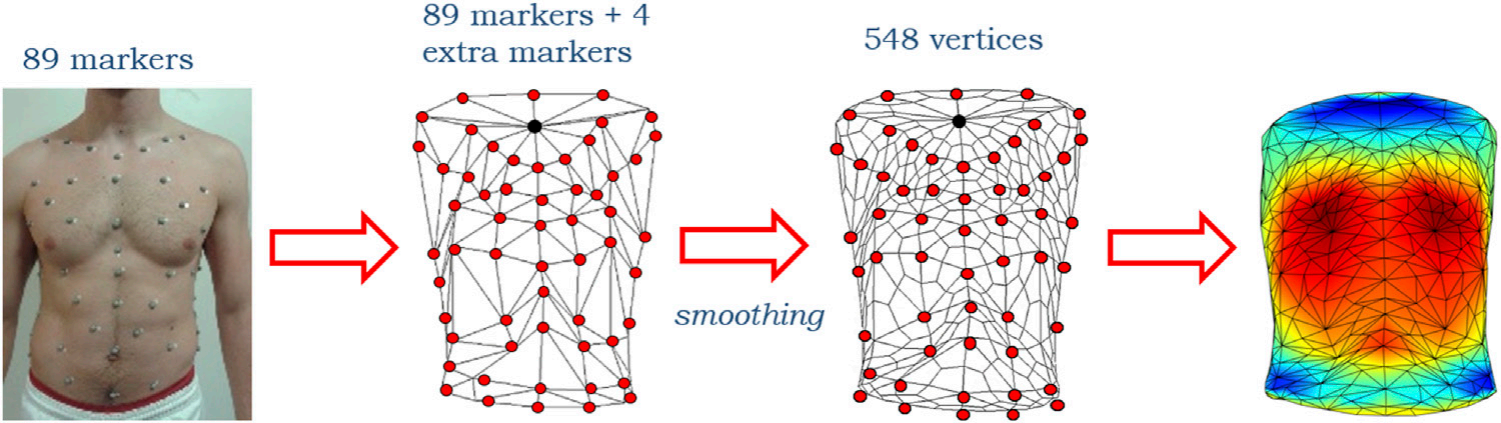
Representative scheme of the initial 89 markers on the subject torso, of the reconstructed 93 markers (89 originals plotted in red plus 4 extra, the upper of which is plotted in black) configuration before and after the smoothing that made the number of vertices increase up to 548 and then a 3D heatmap plot.

Starting from 93 initial markers (89 originals plus 4 extra), the number of vertices after the first smoothing iteration was increased to 548 (Figure 2).

### Models Analysis and Measurements

Once all the 3D smoothed models were obtained, some measurements were performed to assess the geometry of the trunk. 3ds Max features a series of built-in tools for geometrical measurements in terms of linear distances, perimeter, overall surface area, and volume according to the selected object.

By selecting a 3D object in the scene, its volume and surface area are automatically computed by the software and displayed. If the selected object is two-dimensional (e.g., a transversal section of the trunk), 3ds Max automatically computes and displays the area of the section and its perimeter. To measure the linear distance between two points (e.g., two markers) the user has to select the extremes and then the “Measure Distance” tool. 3ds Max automatically displays the value of the distance.

All these features were used to quantify the geometry of the 3D models in terms of: 1) lengths (trunk height, measured along the longitudinal axis between top and bottom central markers; anteroposterior (AP) and mediolateral (ML) diameters at an angle of Louis, xiphoid, and umbilical levels, measured as distances between couples of specific markers; perimeters of the sections at an angle of Louis, xiphoid, and umbilical levels); 2) areas (total chest wall and transversal sections areas at angle of Louis, xiphoid, and umbilical levels created by cutting the model with three planes at the reference heights previously computed) and 3) volumes of the total chest wall and of the three compartments, expressed as absolute values and percentage of the chest wall volume, computed as the ratio between single compartments volumes and their sum. Lengths were measured in centimeters, areas in squared centimeters, and volumes in liters.

All the measurements were performed at four static respiratory volumes: residual volume (RV), functional residual capacity (FRC), end of tidal volume (FRCVT), and total lung capacity (TLC) selected from the total chest wall volume variation.

The differences between all the corresponding quantities listed and measured earlier were also computed, to study the variations in volume. In particular, the difference between values at FRCVT and FRC expressed the tidal volume (VT), the interval between TLC and FRC represented the inspiratory capacity, and the variation between TLC and RV expressed the vital capacity. This step allowed for the evaluation of the ventilator pattern in a quantitative way.

Starting from the previously mentioned parameters, a set of other variables was derived: the ratio between AP and ML diameters, computed at the three reference levels, the surface-to-volume ratio (expressed as cm^−1^), and the ratio between trunk height and perimeter at the xiphoid level. All these variables were considered as a set of shape factors to characterize the trunk’s geometry. Together with geometry measurements, minute ventilation (expressed as liters per minute, L/min) was obtained by multiplying VT and respiratory rate.

### Vector Field Data

The accuracy of the 3D models allowed us to study the kinematics of the breathing pattern. The Morpher modifier in 3ds Max allows to creation a simple and automatic animation by associating two meshes with the same number of vertices. Indeed, by changing a similarity coefficient of the modifier, one mesh can be gradually transformed into a second one. This tool was useful to observe the trunk motion during breathing. A dedicated script was therefore created to build the vector field (VF) of the motion of each vertex of the mesh. The VF was obtained considering the position of corresponding vertices at two different volumes. The direction and modulus of the vector connecting the vertices expressed the amount of motion associated with that precise point of the trunk. The script connected the corresponding vertices of two objects with segments and if they were moved, the segments were automatically adjusted to match the new vertices' positions. Also, the script computed the distance between corresponding vertices (the length of each segment) and determined the direction of the vector. The direction of the vector provided important information because it allowed assessing if the motion was correctly synchronized with the respiratory phases or if presented some paradoxes. The script checked if the endpoint of each vector was either outside or inside the starting volume and, if it pointed inward, added a negative sign to the modulus. The coordinates of starting and ending points of each vector, together with the modulus, were saved into a text file to be further analyzed. The vertices of the faces of the smoothed mesh were also saved to rebuild the model outside 3ds Max. The vector field basically represented the punctual difference between models evaluated at two separate volumes in the respiratory pattern. For each subject, three vector fields were extracted: the first one represented the motion during the quiet breathing inspiratory phase, from FRC to FRCVT that corresponded to tidal volume; the second one was the inspiratory capacity, from FRC to TLC; and the third one captured the maximal expiratory motion from TLC to RV, and represented the vital capacity.

### 3D Heatmap and VF Plot

**FIGURE 3:**
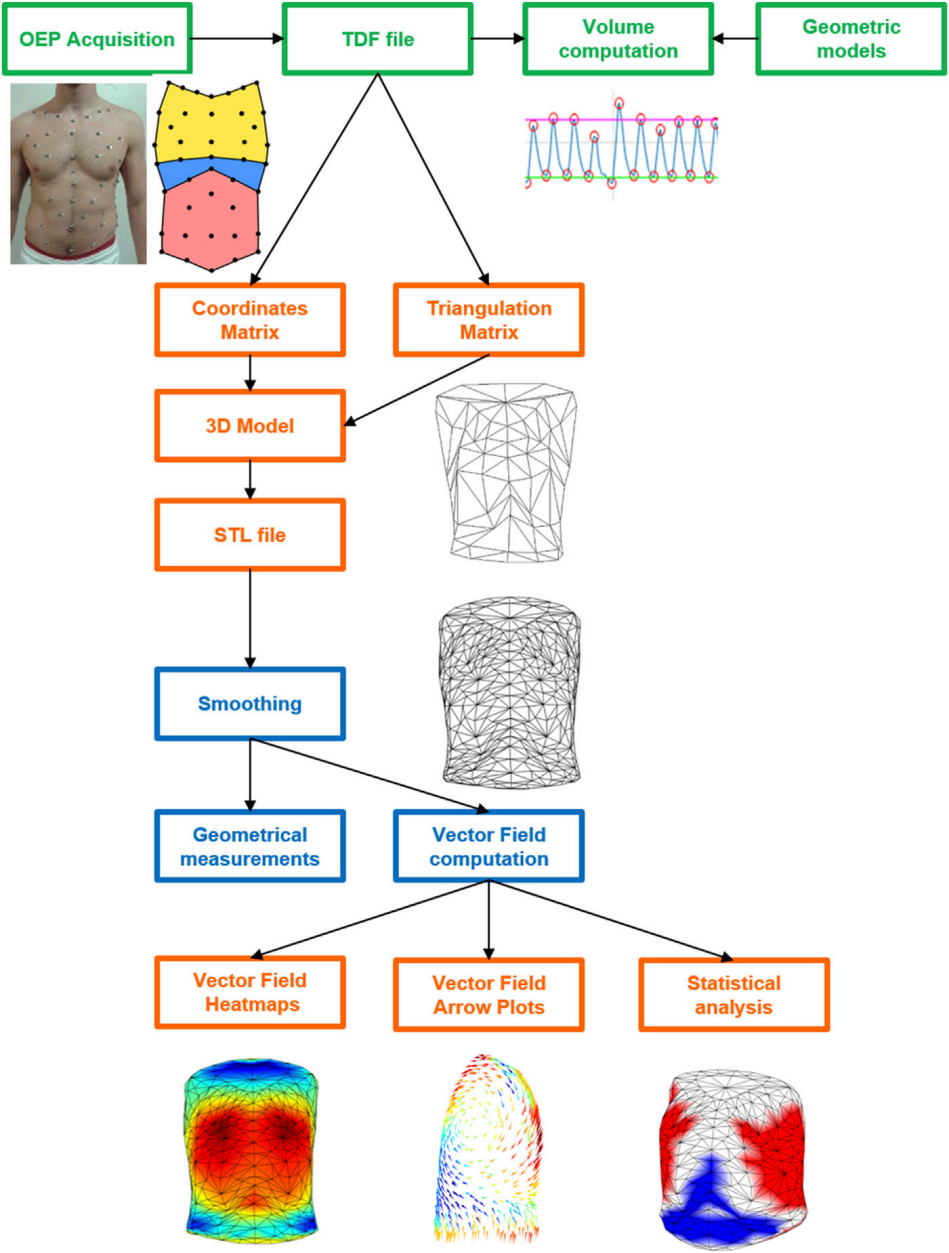
Schematic diagram of the whole process from 3D markers’ coordinates to 3D heatmap and VF plot starting from OEP acquisition (green), passing from the elaboration of the coordinates (TDF file) and triangulation matrixes in MATLAB (orange) to create the 3D models in the STL file that was then elaborated in 3ds Max for the smoothing and the computation of the geometrical parameters and of the vector field (blue) The latter was plotted as colormaps and arrow plot as well as in terms of statistical results in MATLAB (orange).

To better display the information computed, all the data from the text files were then imported in MATLAB. Two kinds of graphs for each VF file were generated: 1) a 3D heatmap that represented the model of the trunk at the starting volume, where colors were associated with the vertices’ motion. The outward (positive) movement was displayed in red, whereas blue areas represented inward (negative) displacement. A color bar was set as a reference for the scale of values (expressed in centimeters, cm) and the range of color; and 2) a 3D VF plot, in which all the vectors were shown as color-coded arrows, with length and direction reflecting the VF data. This particular kind of plot was created using the function arrow3, developed by Jeff Chang and Tom Davis as a free MATLAB plugin function (Chang, 2022). The function was slightly modified because the colormap of the arrows did not consider the sign of the vectors. The magnitude of each vector (with sign) was added to the function inputs to directly map the colors, without computing the difference between starting and ending points. If the user selected files from multiple subjects, other 3D heatmaps were plotted (together with the ones previously described) to compare the different vector fields. However, the VF data had to be normalized first. The lengths of all the vectors belonging to all the selected subjects were saved into a matrix and rescaled between −1 and 1 according to the maximum and minimum values contained in the matrix. To better compare the average displacements, the colormaps were normalized between maximum and minimum VF values. A graphical representation of the whole process from 3D markers’ coordinates to 3D heatmap and VF plot is reported in Figure 3.

### Data Acquisition

The algorithm of analysis was applied to a group of healthy subjects in the seated position. The inclusion criteria were: general good health condition, no respiratory or chronic diseases, no chest wall deformities, age between 25 and 45 years old, and BMI < 26 kg·m^−2^.

First, the subjects were asked to breathe normally for 3 min and then to perform a maximal capacity maneuver, that consisted of a full inspiration followed by a forced full expiration. The markers were attached to the skin according to a standard protocol that defined specific anatomical reference points and used 89 markers (Cala et al., 1996). This configuration covered the entire trunk with a grid of seven circumferential rows and five columns of markers both on the front and on the back.

### Validation

Absolute chest wall volume at FRC, TLC, and RV and chest wall volume variation during quiet breathing, inspiratory capacity, and vital capacity computed on the 3D smoothed models were compared to the corresponding values computed in original OEP 89 markers files considered as the gold standard. Indeed, the OEP volumes were previously validated by comparing them with a flowmeter. The OEP demonstrated a strong correlation during quiet breathing and the vital capacity manoeuver (Cala et al., 1996). OEP was also validated on subjects in the prone and supine positions in healthy subjects (Aliverti et al., 2001). In addition, OEP was also validated in different clinical conditions, namely in intensive care patients (Aliverti et al., 2000) and in infants (Dellaca et al., 2010).

The absolute error was computed as: (OEP 89 markers volume−3D smoothed models volume), while the percentage error was computed as: (OEP 89 markers volume−3D smoothed models volume)/OEP 89 markers volumes*100.

### Statistical Analysis

Not only the geometrical parameters and respiratory variables can be run into comparative analyses between groups, but also the vector field data. Since the normal distribution was not met, the Wilcoxon–Mann–Whitney non-parametric test of hypotheses was applied. In the present work, sex was considered the independent variable. The returned value of *p* indicates that the test rejects the null hypothesis at the 5% significance level. The comparison was performed not only for the geometrical and ventilatory parameters but also for the kinematics of the single triangle of the mesh. In the last case, a vector of *p*-values was therefore obtained for each triangle and used to represent the test results directly on the 3D model separately for each considered volume (namely, VT, IC, and VC). Starting from an averaged and uncolored 3D model obtained from all the subjects belonging to the groups of interest (male vs. female), the points that had a *p*-value below the threshold (5%) were colored: red for points where the first group presented a greater average displacement than the second group, and blue for the opposite situation (Figure 3). All data in the tables are expressed as median, 25th, and 75th percentiles. Absolute values of VF magnitude were considered. The statistical analysis was performed in MATLAB (MathWorks^®^ Inc.).

## Results

### Subjects

Ten healthy subjects (five females and five males similar in age as shown in Table 1) were enrolled among colleagues, relatives, and friends of the authors. All subjects signed informed consent forms. The study got the ethical approval of the Ethics Board of Politecnico di Milano (Parere n. 47/2021) in accordance to the Declaration of Helsinki.

**TABLE 1:** Age and breathing pattern.

	Male			Female			*p*-value
	Median	*p*25	*p*75	Median	*p*25	*p*75	
Age [years]	30.00	29.50	37.75	33.00	28.00	39.25	0.750
RR [breaths/min]	15.00	12.00	18.25	18.00	17.25	21.00	0.136
MV [L/min]	9.00	8.27	11.73	6.92	6.67	9.48	0.175
Tidal volume							
RC,*p* [L]	0.20	0.18	0.40	0.16	0.15	0.19	0.173
RC,*p* [% CW]	40.42	31.64	47.85	38.71	37.62	44.32	0.917
RC,a [L]	0.13	0.12	0.23	0.09	0.05	0.11	0.046
RC,a [% CW]	26.38	19.48	28.93	22.35	14.23	26.67	0.347
AB [L]	0.16	0.16	0.34	0.16	0.14	0.17	0.435
AB [% CW]	36.55	29.60	38.51	38.95	30.35	48.58	0.465
CW [L]	0.48	0.45	0.96	0.41	0.35	0.43	0.028
CW [L]	0.51	0.47	1.02	0.43	0.38	0.47	0.036
Vital capacity							
RC,*p* [L]	1.91	1.17	2.15	1.17	1.00	1.76	0.251
RC,*p* [% CW]	37.73	27.20	47.09	41.05	38.24	47.15	0.465
RC,a [L]	1.35	0.88	1.48	0.63	0.58	0.92	0.028
RC,a [% CW]	28.61	23.24	29.89	22.93	18.75	27.66	0.347
AB [L]	1.61	1.34	1.76	0.99	0.80	1.37	0.047
AB [% CW]	31.75	29.47	44.60	32.15	30.11	36.41	0.917
CW [L]	4.55	4.02	5.07	3.18	2.75	3.45	0.047
CW [L]	4.93	4.31	5.47	3.43	2.98	3.70	0.047

AB, abdomen; CW, chest wall; L, litre; MV, minute ventilation; RC,*p*, pulmonary ribcage; RC,a, abdominal ribcage; RR, respiratory rate.

Two case reports were also considered in a seated position during resting quiet breathing. A 29 years old woman (height: 161 cm) was studied at the 15th (weight: 56 kg; BMI: 21.6 kg/m^2^), 20th (weight: 59 kg; BMI: 22.8 kg/m^2^) and 32nd (weight: 61 kg; BMI: 23.5 kg/m^2^) gestational weeks. A 67 years old man (height: 166 cm; weight: 61 kg; BMI: 22.1 kg/m^2^) before and after a demolition surgery implying, among others, the removal of six ribs on its left size and a soft metallic mesh placed to protect the organs (Marulli et al., 2014), due to thoracic sarcomas.

### Breathing Pattern and Chest Wall Geometry

At rest, during quiet breathing, minute ventilation and respiratory frequency were similar, while male tidal volume was higher because of a higher abdominal ribcage expansion. Similarly, vital capacity was higher in men because of higher expansion of the abdominal ribcage but also of the abdomen. While absolute thoraco-abdominal volumes differ between the groups, no sex-induced dynamic difference in thoraco-abdominal contributions during breathing was found (Table 1). Similarly, at functional residual capacity, the static thoraco-abdominal distribution was similar between men and women, with the latter being characterized by total chest wall lower dimension. The reduced female geometry was mainly in terms of perimeters and areas at all the considered levels, namely Louis angle, xifoideal, umbilicus as well as in terms of the entire chest wall. In addition, women were also characterized by lower volume of the pulmonary ribcage and of the chest wall, by reduced trunk height and lower anteroposterior diameter but also at the level of Louis angle (Table 2).

**TABLE 2:** Chest wall geometry at functional residual capacity.

	Male			Female			*p*-value
	Median	*p*25	*p*75	Median	*p*25	*p*75	
Volume							
RC,*p* [L]	12.1	11.3	13.0	8.2	7.6	8.4	0.009
RC,*p* [%CW]	51.9	49.2	55.2	51.1	48.9	56.8	0.917
RC,a [L]	4.8	3.3	5.4	3.1	2.4	3.2	0.076
RC,a [%CW]	22.2	13.2	22.9	18.7	15.5	21.6	0.602
AB [L]	5.6	5.2	8.7	3.8	3.5	5.3	0.076
AB [%CW]	25.9	24.8	32.2	27.4	24.9	32.2	0.917
CW (Sum) [L]	21.7	21.1	26.9	14.7	13.4	16.5	0.009
CW (Obj) [L]	23.2	22.3	28.5	15.6	14.2	17.7	0.009
Trunk height [cm]	42.5	40.4	44.3	36.7	36.3	37.7	0.009
Diameter							
AP louis angle [cm]	19.8	19.1	21.7	16.9	16.3	18.0	0.009
AP xifoideal [cm]	23.2	22.0	24.6	21.6	19.0	22.9	0.175
AP umbelicus [cm]	22.7	21.5	27.4	20.1	18.9	21.7	0.076
ML louis angle [cm]	27.7	26.1	29.0	25.5	22.0	26.3	0.076
ML xifoideal [cm]	27.5	26.2	29.8	25.2	21.6	27.4	0.175
ML umbelicus [cm]	27.4	26.7	31.2	26.3	23.0	27.4	0.076
Perimeter							
Louis angle [cm]	92.2	91.4	98.7	80.9	78.5	82.1	0.009
Xifoideal [cm]	90.1	86.7	98.9	79.7	76.3	84.4	0.028
Umbelicus [cm]	82.5	81.4	96.6	76.5	71.3	82.3	0.047
Area							
Louis angle [dm^2^]	6.1	5.8	6.7	4.4	4.3	4.6	0.009
Xifoideal [dm^2^]	6.0	5.7	7.2	4.8	4.3	5.2	0.028
Umbelicus [dm^2^]	5.1	5.0	7.2	4.4	3.9	5.1	0.047
CW [dm^2^]	45.6	44.7	52.2	35.3	33.8	38.9	0.009

AB, abdomen; AP, anteroposterior; CW, chest wall; L, litre; ML, medio-lateral; RC,*p*, pulmonary ribcage; RC,a, abdominal ribcage.

### Validation

The median error between OEP 89 markers files (gold standard) and the 3D smoothed models in terms of absolute volumes were: 0.97 L at FRC; 1.04 L at TLC; 0.81 L at RV. The median error between OEP 89 markers files (gold standard) and the 3D smoothed models in terms of volume variations were: −11 ml for the tidal volume; 62 ml for the inspiratory capacity and 169 ml for the vital capacity.

The median percentage error between OEP 89 markers files (gold standard) and the 3D smoothed models in terms of absolute volumes were: 4.5% at FRC; 4.3% at TLC; 4.2% at RV. The median percentage error between OEP 89 markers files (gold standard) and the 3D smoothed models in terms of volume variations were: −2.6% for the tidal volume; 2.4% for the inspiratory capacity and 3.7% for the vital capacity.

### Colormaps and Vector Field Analysis

**FIGURE 4:**
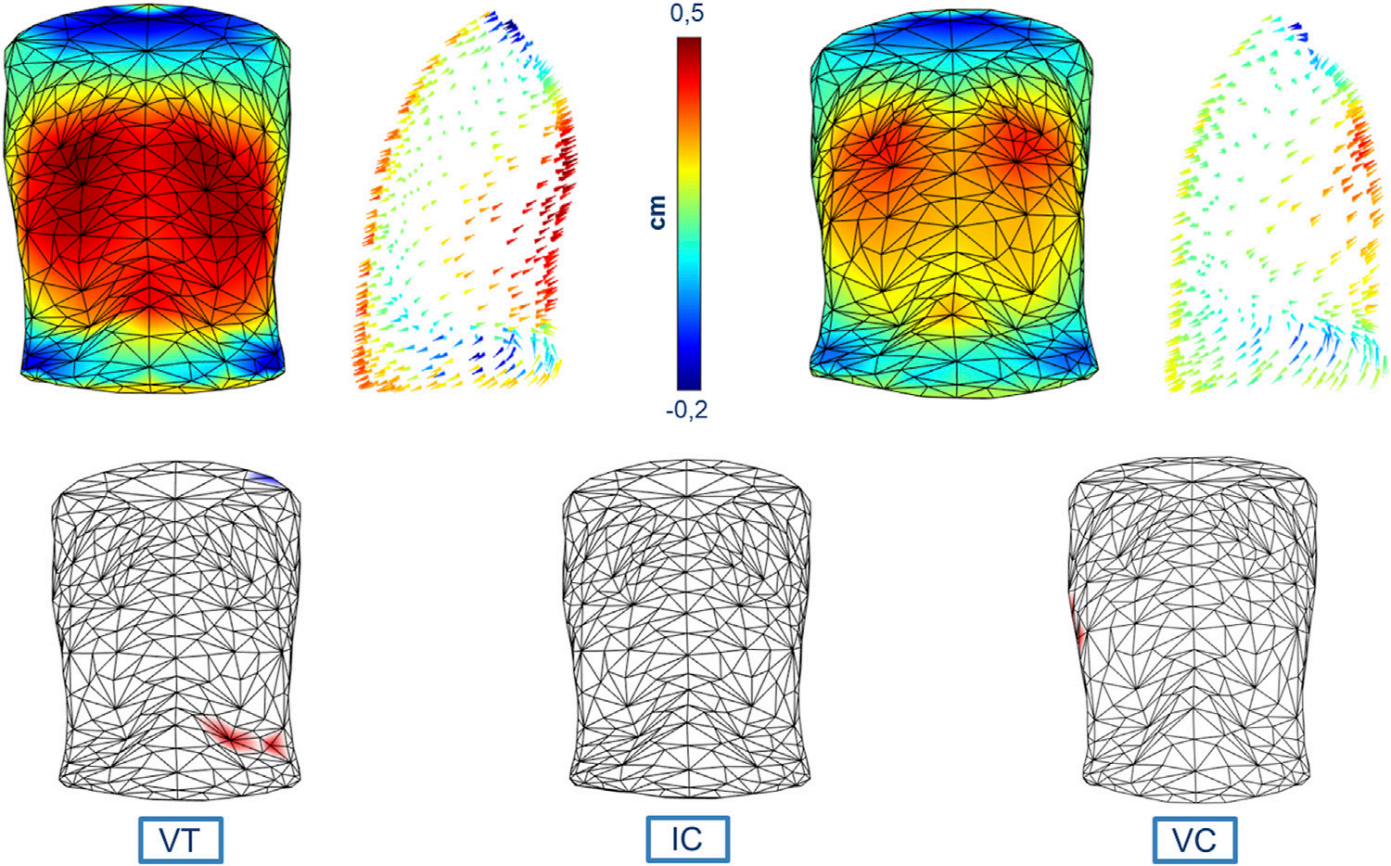
Colormaps of the magnitude and vector direction of the average trunk motion during tidal volume in healthy men (top left panels) and healthy women (top right panels). The statistical colormaps (bottom panels), that highlight the most relevant differences between respiratory patterns, are reported on an averaged and uncolored 3D model obtained from all the subjects, with the points that had a *p*-value < 0.05 colored (red when males presented a greater average displacement than females, and blue for the opposite situation) for each of the three ventilatory phases considered: tidal volume (bottom left panel, VT), inspiratory capacity (bottom middle panel, IC), and vital capacity (bottom right panel, VC). The uniform color scale is also reported (top middle panel).

Figure 4 reports both the magnitude and the direction of the average trunk motion shown through specific colormaps that have uniform color scales to better compare the results. The statistical colormaps, that highlight the most relevant differences between respiratory patterns, are also reported. The significance level was set at 95% (*p*-value < 0.05). For each comparison, three ventilatory phases were considered: Tidal Volume (VT), Inspiratory Capacity (IC), and Vital Capacity (VC). No significant differences were found between healthy men and women even if the overall magnitude of the displacement was slightly greater in men (as indicated by the colors).

## Discussion

A new non-invasive and accurate method for respiratory analysis was successfully developed and proposed. This method allowed for the creation of realistic 3D models of the trunk that offered an accurate and complete description of the thoraco-abdominal surface geometry and kinematics during respiration at two levels. Accurate local motion analysis as well as global geometry assessment of the thoraco-abdominal surface can be processed. The latter was derived by the anthropometric analysis and it was useful to study the overall trunk shape and its compartments; the former was extracted from vector field data, representing the field of motion of each point. The colormaps and the vector field analysis are graphical outputs immediate to be interpreted that potentially candidate the proposed method to be easily translated into clinical practice.

The described procedures were implemented using a general and flexible approach in order to obtain a versatile and repeatable analysis method. This was one of the most important strengths of the proposed method together with the standardization of the outputs that allowed the comparison among different subjects and different volumes. In this way, it was possible to characterize the shape of the trunk with a very small spatial resolution using non-ionizing radiations as well as to derive static and dynamic (i.e., due to breathing) maps of the local motion of the chest wall. These characteristics are very important since they allowed a multitude of possible future physiological, pathophysiological, and clinical applications.

Although this was principally a methodological study, a first example was proposed on a small group of subjects to verify the effectiveness of this new approach. We deliberately chose to start with a pilot study on healthy, normal-weighted subjects of similar age to try to reduce as much as possible confounding factors, like scoliosis, other deformities, obesity or diseases, that could have influenced the results of the analysis.

Taking into account all the quantities that have been measured (volumes, areas, perimeters, and diameters), it was possible to notice several differences among healthy controls, with sex playing a role, particularly in terms of some geometrical and expansion parameters.

Despite the small sample size, the differences between healthy males and females reflected the results shown by Romei et al. (2010). We started with the study of sex influence on anatomical and respiratory function parameters because sex is recommended of being included as a factor in clinical practice norms and as a topic of the bench and clinical research. Sex is recognized to play an important role in respiratory physiology according to two pathways: hormones and anatomy. The former takes part in lung inflammatory processes, in control of breathing and in response to diseases; the latter distinguishes females for being characterized by smaller dimensions at every level of the respiratory system (García-Martínez et al., 2016; LoMauro and Aliverti, 2018; Torres-Tamayo et al., 2018; LoMauro and Aliverti, 2021). Of course, future studies should be aimed to include a greater number of subjects in order to give the possibility to perform other comparative analyses, for example, by considering combined features other than sex such as different ages and weight. Another important parameter that may be included in future studies could be the variation of the posture of the subject. Postural change plays a role, particularly in the function of thoracic and abdominal muscles, which is then reflected in thoraco-abdominal volume expansion (Romei et al., 2010).

The results from the colormaps and vector field analysis showed how the influence of sex on the breathing pattern is not visible on a local scale (point to point), but it can be assessed at the compartmental level, as stated in previous studies (Romei et al., 2010). This result was somewhat expected considering the choice to analyze healthy subjects of similar age, but also from the low number of the population. The aim of the present study was to present and describe the new method and its potential and not to show differences between sexes on the ventilatory and thoraco-abdominal pattern. To do so we would have needed to correct for multiple testing, since ventilatory and thoraco-abdominal pattern comprises different parameters. Without this correction, the statistical significance would have been overestimated (Groenwold et al., 2021). We performed an “exploratory” analysis, with the results being limited to the single parameter and not to a more generalized sex effect on breathing. Similarly, for the 3D maps statistical parametric mapping and random field theory for the analysis of data would be implemented to derive general conclusions and/or to avoid the error propagated over the big number of tests performed.

**FIGURE 5:**
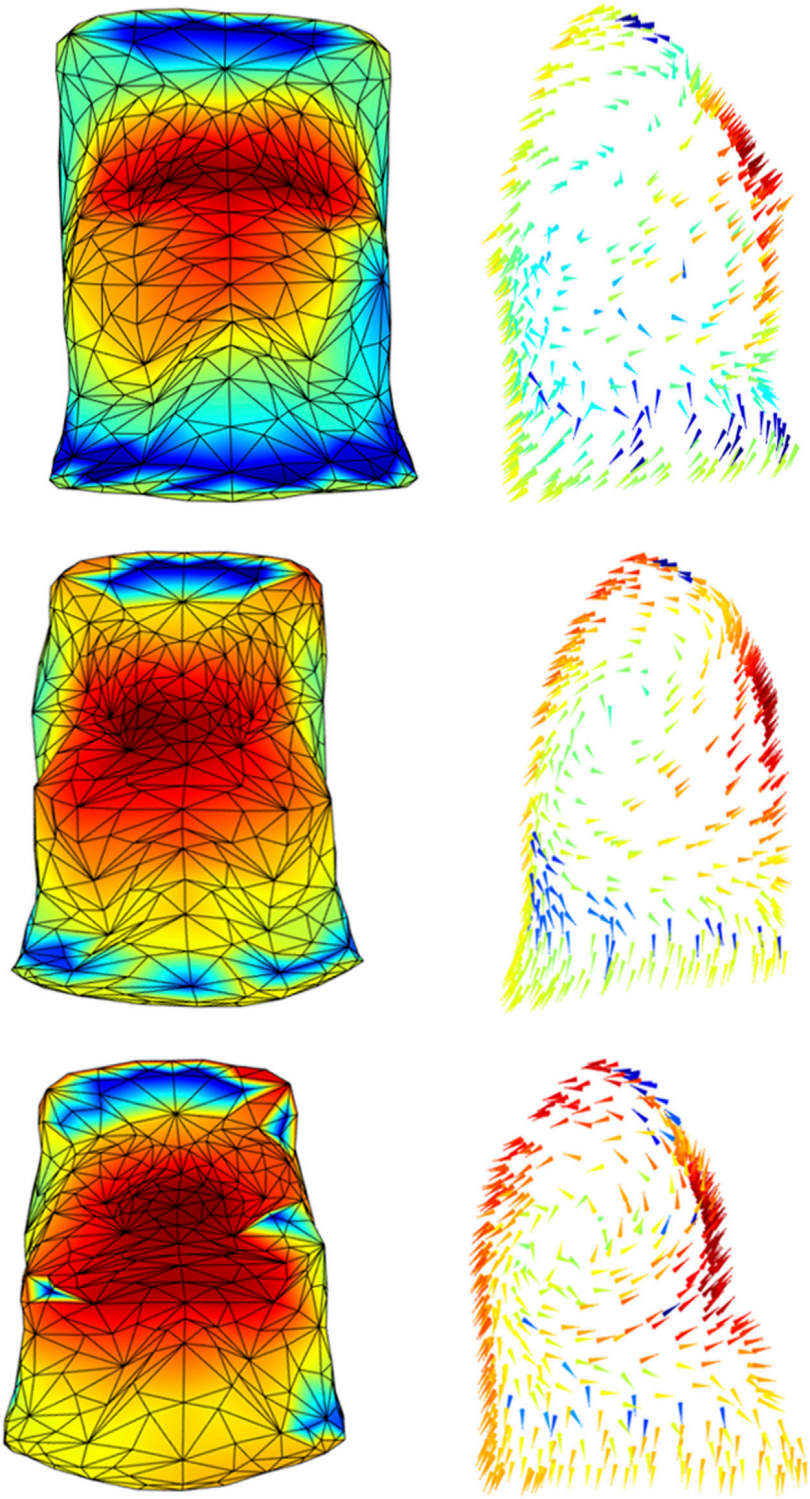
Colormaps of the magnitude (left panels) and vector direction (right panels) of the trunk motion during the tidal volume of a woman studied at the 15th (top panels), 20th (middle panels), and 32nd (bottom panels) gestational weeks. In line with the literature, the progression of pregnancy breathing was associated with increased abdominal expansion (from azure-blue of the top panel to yellow-red of the bottom panel).

We believe that this method could provide important additional information for the physiopathology of diseases implying asymmetric or paradoxical breathing as well as in the case of local thoraco-abdominal changes. To support this idea, we presented two qualitative examples. Figure 5 showed how the method was able to track the pregnancy-induced changes in a woman chest wall. In this case, the woman was studied during each trimester of pregnancy (LoMauro et al., 2019). She gained only five kilos of weight, completely located in the abdomen. In addition to the expected changes in abdominal dimensions, the figure immediately showed how she shifted her breathing towards the abdomen. In seated position resting breathing is predominantly thoracic (Romei et al., 2010), with the abdomen expanding not much as indicated by the blue area/arrows during the first trimester of gestation. With the progression of pregnancy, the abdominal area/arrows became progressively yellow therefore indicating an increased abdominal contribution to tidal breathing (Figure 5). This was confirmed by the analysis of the volume variation and it was interpreted as an additional contribution of the diaphragm that it is put in mechanical advantage, because of the stretching effect of the enlarging uterus (LoMauro et al., 2019).

**FIGURE 6:**
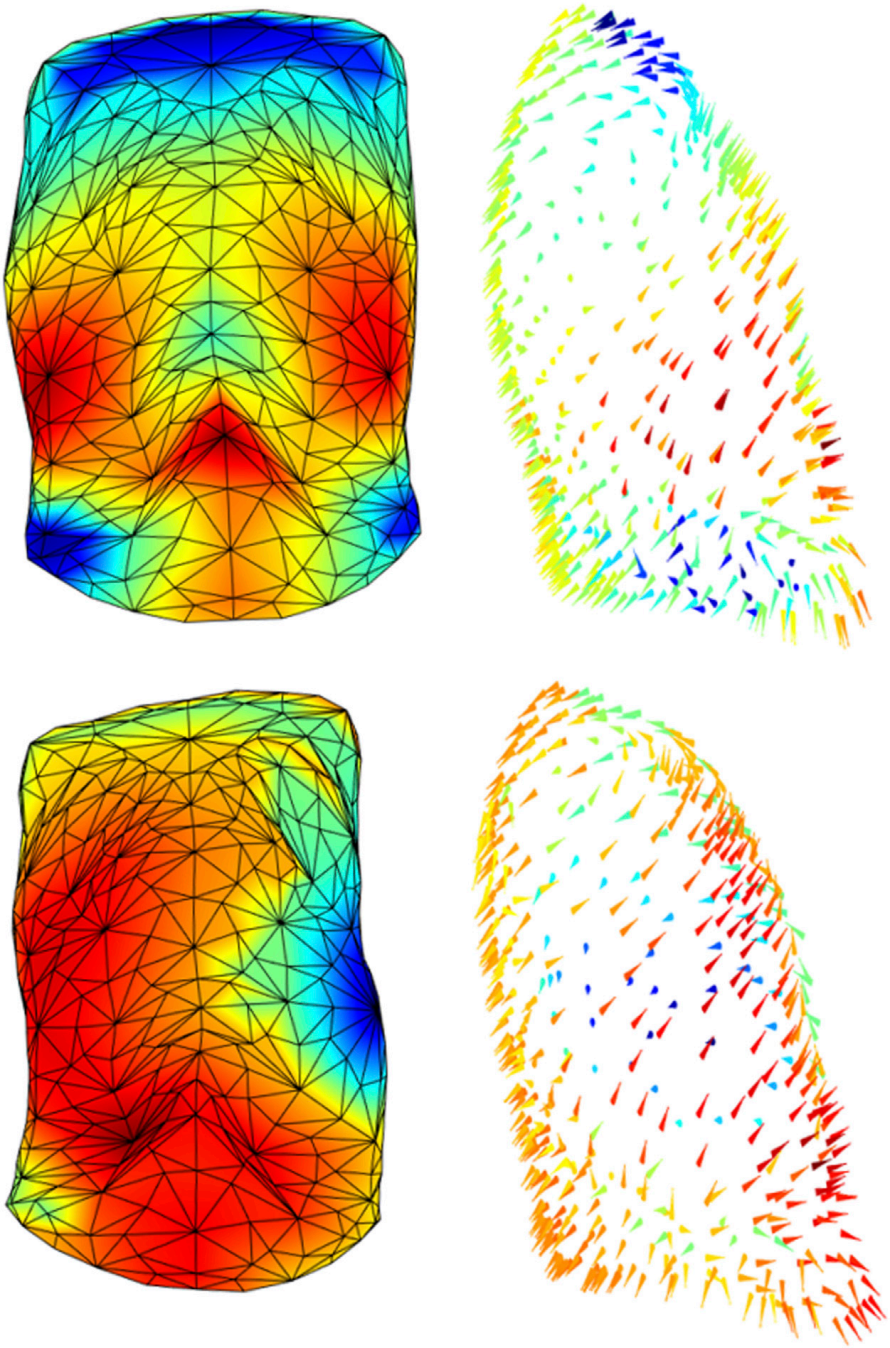
Colormaps of the magnitude (left panels) and vector direction (right panels) of the trunk motion during the tidal volume of a patient before (top panels) and after (bottom panels) a demolition surgery of his left ribcage. Since the surgical procedure was inevitably tailored to the patient, the impact on the chest wall does not follow a strictly “compartmental” logic but it was more diffused among compartments, rather than confined to a “specific” compartment. It is also evident that patient passed from an almost perfectly symmetric breathing expansion to a paradoxical inward inspiratory movement of his left side (blue triangles, bottom panel).

By contrast, Figure 6 showed a patient before and after the removal of six ribs on his left size. The patient passed from a pre-surgical symmetric expansion to an inward inspiratory paradoxical movement of the corresponding demolished portion of the ribcage (blue area/arrows). It was also evident that a general reorganization of the chest wall expansion occurred with also an increased abdominal expansion. This is a dramatic example of the potentiality of the proposed system because it allowed to identify possible altered patterns of expansion in every point of the chest wall, therefore, overcoming the concept of the anatomical compartment.

These two extreme and opposite case series, the first implying localized and symmetric abdominal expansion and the second asymmetric and asynchronous ribcage expansion, encourage the potential clinical translation into clinics of the proposed method. In addition, they proved how the graphical representation was immediate in highlighting the significant changes in chest wall expansion. Of note, such qualitative immediate output is paralleled with rigorous analytical quantification of local expansion and relative changes.

In addition, the methods presented can also be applied to other marker configurations. For instance, analyses performed on children are typically based on a lower number of markers because of the small pediatric thoracic size. If the same markers’ size (generally 1 cm of diameter) and number (generally 89 in seated and 52 in supine position) used for adults are applied to children, there might be the risk that markers would be too close to be distinguished by the OEP system. There are two solutions to solve this problem according to the equipment available in the single laboratory: 1) to use smaller markers without reducing the number of markers used in adults and/or 2) to zoom in the TVC cameras of OEP system.

The process of creation of the 3D models can be easily adapted to other acquisition protocols. More in general, the local shape analysis procedure could be set up for other pathological conditions that have an impact on the trunk geometry and/or on respiratory muscles. As shown in Figure 1, all three parts of the chest wall might paradoxically move during inspiration, being a consequence of an imbalanced action between the ribcage muscles and the diaphragm. The sternum deformity in severe Osteogenesis Imperfecta, together with severe scoliosis, put the ribcage muscles at mechanical disadvantage so that they become unable to counteract the action of the diaphragm resulting in a pulmonary ribcage paradoxical inward (LoMauro et al., 2012). By contrast, when the diaphragm is paralyzed (like in severe glycogen storage disease) the abdomen is passively under the action of the sub-atmospheric pressure generated by the contraction and therefore it moves paradoxically (Remiche et al., 2013). The analysis based on compartments, although able to identify asynchrony and asymmetry (LoMauro et al., 2021), does not allow highlighting anomalies between and within compartments that occur in specific pathological conditions. For this reason, the local analysis provided by this new method would be highly informative with the potential ability to track and quantify the progression of degenerative disease and/or the efficacy of a specific therapy. These might comprise thoracic surgery, with pulmonary complications being a major cause of morbidity and mortality in the postoperative period. The type and severity of such complications depend on the performed thoracic surgery, with the surgical approach playing a crucial role, as well as on the patient’s pre-operative medical status. Thoracic surgery inevitably impacts chest wall kinematics by restricting its expansion (Nosotti et al., 2010; LoMauro et al., 2017; Tukanova et al., 2020). The restriction might be strongly related to accessing the intrathoracic organs through the bony ribcage. This ranges from more invasive open thoracotomy, which implies larger incisions, stretching across the chest, dissection of and trauma to large muscles, to minimally invasive surgery, which minimizes the overall surgical trauma suffered by the patient (Wong et al., 2018). Of course, the surgical procedure is inevitably tailored to each patient and therefore the impact on the chest wall does not follow a strictly “compartmental” logic. Figure 6 strongly supported this idea, as it showed and quantified how the impact of surgery on thoracic expansion was more diffused among compartments, rather than confined to a “specific” compartment. For these reasons, comparing the 3D pre-operative to the 3D post-operative models not only might help localize the surgically induced restriction but also to track the effectiveness of rehabilitative interventions aimed to reverse patients’ complications and recruiting lung/chest wall expansion.

Another important and widespread clinical condition that might benefit from 3D models is thoracic deformities that involved the ribcage alone, the spine alone, or both together. Severe spinal deformities, like scoliosis, are known to induce lung function impairment (Lin et al., 2001; Szopa and Domagalska-Szopa, 2017). Severe Osteogenesis Imperfecta is characterized by both sternum and spinal deformity (LoMauro et al., 2012), leading to an important lung restrictive pattern (Storoni et al., 2021). The 3D modeling of chest wall expansion can therefore help tracking the impact of any kind of intervention (either surgical or bracing or rehabilitative) of spinal deformities in terms of improvement of the breathing function. Since the thoracic deformities differ among patients and are distributed along the entire thorax, local analysis is preferable to have a precise characterization of the deformity itself and of the effectiveness of the treatment.

Considering the study of trunk kinematics, the aforementioned analysis techniques can be extended to other fields as well. The combination of optoelectronic tracking and kinematics analysis can enhance some particular features in any kind of motion. For example, it could be useful to investigate specific movements performed by athletes. By recording the motion and studying the vector field of each point, new information can be extracted in order to improve their performance because all the measured quantities could be potentially correlated in order to find any significant relationships between respiratory and motor function.

Finally, the accuracy of the system was below 5% in both all the considered volumes, with the absolute error being in the order of ∼1 L for absolute chest wall volume and a few ml for volume variations. Our results suggest that meshing the surface with a higher number of points and a higher number of triangles, that is, considering an approximation closer to the real surface, provides slightly lower volumes and volume variations than approximating the surface with 89 markers. Although further studies are needed to fully demonstrate this effect, it is important to underline that uncertainty of 5% is likely to be lower than the level of clinical significance, particularly for volume variations (i.e., 11 ml uncertainty on tidal volume is negligible for an adult).

To conclude, starting from the 3D coordinates of markers put on the chest wall, it was possible to create realistic and reproducible 3D dynamic models of the trunk for immediate graphical interpretation. The methods presented were able to confirm the results obtained in previous works and proposed some new insights about trunk geometry and ventilator kinematics analysis with a high level of accuracy. This might have important physiological, pathophysiological, and clinical implications as the chest wall motion is a consequence of respiratory muscle contraction as well as respiratory mechanics.

## Data Availability

The raw data supporting the conclusion of this article will be made available by the authors, without undue reservation.
